# Assessment of the Autism Spectrum Disorder Based on Machine Learning and Social Visual Attention: A Systematic Review

**DOI:** 10.1007/s10803-021-05106-5

**Published:** 2021-06-08

**Authors:** Maria Eleonora Minissi, Irene Alice Chicchi Giglioli, Fabrizia Mantovani, Mariano Alcañiz Raya

**Affiliations:** 1 Institute for Research and Innovation in Bioengineering (i3B), Universitat Politécnica de Valencia, Ciudad de la Innovación, Building 8B, s/n Camino de Vera, 46022 Valencia, Spain; 2 Department of Human Sciences for Education ‘‘Riccardo Massa’’, University of Milano Bicocca, Milan, Italy

**Keywords:** Autism spectrum disorder, Machine learning, Eye tracking, Social visual attention, Assessment, Classification

## Abstract

The assessment of autism spectrum disorder (ASD) is based on semi-structured procedures addressed to children and caregivers. Such methods rely on the evaluation of behavioural symptoms rather than on the objective evaluation of psychophysiological underpinnings. Advances in research provided evidence of modern procedures for the early assessment of ASD, involving both machine learning (ML) techniques and biomarkers, as eye movements (EM) towards social stimuli. This systematic review provides a comprehensive discussion of 11 papers regarding the early assessment of ASD based on ML techniques and children’s social visual attention (SVA). Evidences suggest ML as a relevant technique for the early assessment of ASD, which might represent a valid biomarker-based procedure to objectively make diagnosis. Limitations and future directions are discussed.

## Introduction

Autism Spectrum Disorder (ASD) is a neurodevelopmental disorder affecting worldwide 1 in 160 children (WHO, 2019) which emerges in childhood and persists in adulthood. ASD is defined by the presence of impairments in social interaction and communication, and repetitive and restrictive patterns of behaviours and interests (APA, 2013). Several aspects might contribute to the ASD manifestation, such as neurobiological, genetic, environmental and cognitive factors (Currenti, 2010; Klin & Mercadante, 2006). Although ASD signs may be visible in early childhood (Lord et al., 2006), ASD diagnosis is usually made 2 or 3 years after the appearance of symptoms, at the average age of 4 (Goldstein & Ozonoff, 2018). The stress on social impairments and atypical language development in ASD stems from its scientific evidence (e.g., Dawson et al., 2004; Mundy et al., 1986; Naber et al., 2008; Tager-Flusberg et al., 2005; Wilkinson, 1998), and it might be due to a reduced attention to both social stimuli (SS) and social interaction (Chevallier et al., 2012; Dawson et al., 2005). ASD social impairments include among others eye contact avoidance, altered joint attention (JA), difficulties in social and emotional judgment, social smiling absence, and atypical turn taking (Tager-Flusberg et al., 2005; Wilkinson, 1998). In particular, children with ASD tend to show reduced social visual attention (SVA) to the eyes compared to typical developmental (TD) children (e.g., Sterling et al., 2008; Tanaka & Sung, 2016), as well as deficit in JA related to following eye gaze, pointing towards objects or both (e.g., Dawson et al., 2004; Mundy et al., 1986; Naber et al., 2008), and difficulties in the social evaluation of interlocutor’s kindness (e.g., Wallace et al., 2010, 2017). They also strive for understand and follow turn taking in dialogues, resulting in excessive ASD verbosity (e.g., Chuba et al., 2003; Ghaziuddin & Gerstein, 1996), and they miss social smiling expression in response to the interlocutor’s smile, which is related to the incapacity of both orienting eye gaze and showing positive facial expressions (e.g Dawson et al. 1990; Kasari et al. 1993; Swettenham et al. 1998).

### Traditional ASD Assessments of Social Skills and Diagnosis: Advantages and Limitations

Despite the wide offer of measures to assess ASD, semi-structured interviews and observations such as the ADOS (the Autism Diagnostic Observation Schedule, ADOS; Lord et al., 1999) and the ADI-R (the Autism Diagnostic Interview-Revised, ADI-R; Lord et al., 1994), are considered as the gold standard for ASD assessment in clinical settings (Goldstein & Ozonoff, 2018; Kamp-Becker et al., 2018). ADOS is a sequence of semi-structured observational tasks in which the examiner evaluates child’s responses to several familiar and unfamiliar situations, observing whether ASD-related behaviours occur. On the other hand, ADI-R is a semi-structured interview addressed to family caregivers whose aim is to detect ASD by the interpretation of parents’ reports concerning children’s daily life. Regarding the assessment of social skills, ADOS includes many subtasks which objective is the direct evaluation of social competences, as eye contact, JA, and social smiling; conversely, ADI-R evaluation is based on caregiver reports, and therefore not gives the opportunity to directly observe social behaviours of children as in the ADOS-2. An additional measure to assess ASD is the Child Autism Rating Scale (CARS; Schopler et al., 1980), which is analogous to ADOS since it is based on the clinician’s evaluation of child’s behaviours, following two short rating scales. Moreover, ASD social impairment can be assessed by the Social Communication Questionnaire (SCQ; Rutter et al., 2003), and the Social Responsiveness Scale (SRS; Constantino & Gruber, 2005). Since SCQ and SRS refer to caregivers, they share the same ADI-R limitation regarding the absence of a direct observation on child’s social behaviours. SRS and SCQ have been widely used in research for quick and sharp diagnosis and less in clinical settings, where traditional measures received the greatest consensus. Although traditional measures benefit from good mutual agreement and reliability (e.g., De Bildt et al., 2004; Le Couteur et al., 2008), they present some limitations regarding objective measurement and ecological validity, which arise questions about their real effectiveness (Alcañiz Raya, Chicchi Giglioli, et al., 2020; Alcañiz Raya, Giglioli, et al., 2020; Alcañiz Raya, Marín-Morales, et al., 2020; Goldstein & Ozonoff, 2018). Traditional assessment scores rely on the examiner’s interpretation of respectively child’s behaviours and parents’ reports, hence examiner’s strong expertise in the ASD field, as well as clinical training in ASD assessment procedure, are highly recommended to avoid the misleading detection and interpretation of symptoms (Lord et al., 2001; Reaven et al., 2008). However, methodological limitations in the ASD traditional assessment rely on measured variables as well, and not only on examiner’s expertise and training. Tapped variables in current ASD assessment represent the behavioural presence or absence of explicit symptoms, and not the objective evaluation of behavioural symptom underpinnings. In addition, social desirability bias (Paulhus, 1991) could affect truthfulness of responses over the assessment of both children and family caregivers, since they might act or report symptoms differently from what is expected, in the attempt of being perceived as favourable by others. Finally, traditional ASD assessment is not always sensitive to differential diagnosis, as in the case of low-functioning children, who are often wrongly diagnosed as ASD instead of children with intellectual disability (De Bildt et al., 2004).

Regarding ecological validity, it refers to the power of a setting to evoke everyday experiences and realistic behaviours, even though it is not the real world (Franzen & Wilhelm, 1996). The current ASD assessment takes place in neutral settings requiring ecological validity (i.e., laboratory) that neither reflect performance in real life, nor allow generalization of results (Chaytor et al., 2006; Parsons, 2016). The lack of ecological validity in laboratory is because it is a highly controlled setting, wherein is difficult providing the illusion of being in the real world. On the other hand, naturalistic environments allow study observations as if subjects were experiencing everyday life situations, even though they offer cost disadvantages.

Considering these limitations, the underlying issue stems from the lack of objective and ecological measures in ASD assessment, which could provide a more accurate and sensitive ASD diagnosis through the evaluation of specific biomarkers (Alcañiz Raya, Chicchi Giglioli, et al., 2020; Alcañiz Raya, Giglioli, et al., 2020; Alcañiz Raya, Marín-Morales, et al., 2020). Objective and integrative psychophysiological measures related to disorder cognitive and neurobiological correlates, as well as more controlled procedures, and standardized realistic tasks are necessary to improve validity and efficiency of current ASD assessment.

### Eye Movements as Biomarker in ASD Assessment: How to Overcome the Quantitative Method Need

Social cognitive neuroscience was the first scientific field prompting the idea that social interaction is mostly driven by implicit processes far from conscious awareness (Forscher et al., 2019; Lieberman, 2010); therefore, the traditional idea of social cognition models that humans can correctly analyse their behaviours and report their feeling and beliefs is outdated (Nosek et al., 2011). In the last decades, the advances in ASD research pointed out the underlying pathophysiology of ASD and identified possible disorder biomarkers (Bölte et al., 2016). Implicit processes, or biomarkers, represent biological signs in response to either external stimuli or internal processing that can be accurately measured and reproduced (Strimbu & Tavel, 2010). Recent evidences suggest that biomarkers might implement assessment procedures, facilitating early diagnosis, since they represent unconscious brain processes that can objectively disclose ASD (Alcañiz Raya, Chicchi Giglioli, et al., 2020; Alcañiz Raya, Giglioli, et al., 2020; Alcañiz Raya, Marín-Morales, et al., 2020; Klin, 2018; Walsh et al., 2011). The need to find a quantitative method to assess ASD yielded novel challenges to researchers, mirrored by the exponential increase of published studies involving implicit measures in children with ASD (Bölte et al., 2016). Implicit measures that allowed to define ASD biomarkers include among others body movement, neural correlates and activations, electro-dermal activity (EDA), genomics, and eye movements (EM) recording (Choueiri & Zimmerman, 2017; Crippa et al., 2015). For instance, children’s impaired eye gaze in social situations is related to atypical development, in particular to ASD (Chita-Tegmark, 2016); whereas, regarding neural correlates and activations, EEG studies on resting state activity showed that compared to TD children, peers with ASD have reduced network connectivity, as well as reduced power in the alpha frequency range (Matlis et al., 2015). In addition, fMRI studies reported in children with ASD abnormalities in early brain growth and increased white matter volume in several brain regions (Ismail et al., 2016). Finally, body movement analysis of upper limb movements (i.e., hidden fluctuations) recorded by electromagnetic sensors in a basic pointing task allowed to distinguish ASD from TD peers (Wu et al., 2018). Despite the growing impact in literature of implicit measure application to define ASD biomarkers for assessment and intervention, none yet has been validated for clinical use (Walsh et al., 2011). ASD biomarkers might completely change assessment process, and besides, they might reliably track illness progression and personal variation in symptom severity. In particular, EM seems to be promising due to low-cost efficiency and the feasibility of studying infants and young children’s internal cognitive processes with a non-intrusive method (Bölte et al., 2016; Klin, 2018). EM are measured by eye-tracking technology, which is based on infrared cameras recording images of the eye at several customized frequencies. The most studied EM are saccades and fixations: saccades are rapid EM that redirect gaze, which can occur up to four time a second and present a variety of amplitudes, duration and peak velocity; whereas fixations are the EM between saccades in which gaze is stationary and visual information is decoded. In this review, only studies focusing on aforementioned EM in children with ASD and TD peers have been considered. Usually, EM analysis is based on the areas of interest (AOIs) approach, which consists in defining either a priori or a posteriori boundaries in the stimulus in order to analyse specific EM behaviours in different regions. The more traditional a priori AOI approach is a top-down method that generates AOI boundaries according to the semantic parsing of the stimulus (i.e., mouth, eyes, nose and background) and without a statistical reason (Yi et al., 2014). On the other hand, a posteriori AOIs follow a bottom-up approach that avoids to determine AOI size and location, providing more objective outcomes than a priori AOIs, due to an algorithm that considers EM on the entire SS (Cilia et al., 2019a, 2019b). EM research shed light on how people with ASD sample and process social visual information compared to TD peers. Two recent reviews and meta-analysis on SVA in people with ASD and TD (Chita-Tegmark, 2016; Frazier et al., 2017) revealed, on one hand, that individuals with ASD spend less time looking at SS than TD (mean effect size: 0.55; Chita-Tegmark, 2016) and, on the other, that the ASD social impairment is stronger when social complexity in SS increased, from social images (SIs) on faces, where eyes AOI produces the largest effect size (Hedge’s g = 0.47; Frazier et al., 2017), to more complex comparisons of social videos (SVs) and non-social videos (NSVs). ASD SVA seems to be modulated by social content and researchers should try to discern which stimuli may provide specific impairments, taking into account both ASD severity subgroups and rigorous methodology (Chita-Tegmark, 2016). Aforementioned conclusion of Chita-Tegmark (2016) and the quantitative method need in ASD assessment inspired researchers to implement biomarker-based attempts of ASD early discrimination involving children’s SVA and machine learning (ML) techniques.

### ML as Statistical Approach for ASD Assessment

Two main statistical approaches to science exist: explanatory strategy, that tries to describe phenomenon casual underpinnings (i.e., descriptive and inferential statistics), and predictive strategy, that attempts to forecast events that have not been observed yet (Yarkoni et al., 2017). Following a statistical perspective, the model that closely estimates the data-generating process (i.e., data-contingent phenomenon explanation) is not the most successful at predicting realistic conclusions (Shmueli, 2010). Therefore, the current replication study crisis and the consequent need in experimental psychology to move from explanatory strategies towards more predictive strategies have led researcher to consider as fruitful new algorithm-based statistical approaches, such as ML (Orrù et al., 2020; Yarkoni et al., 2017). ML approach can better deal with the statistical explanatory strategy issue of overfitting, which is the propensity for traditional statistical models to mistakenly consider sample-specific noise as if it were relevant (Yarkoni et al., 2017). ML is a subset of artificial intelligence that can be defined as the study of computer algorithms that improve automatically through experience (Mitchell, 1997). ML can be broadly organized into two categories: supervised and unsupervised learning (Mello & Ponti, 2018). The former is the most used in human behaviour research and it is the learning process in which ML received prelabelled data as input (i.e., diagnosis) and use them to predict target classification. On the other hand, the latter ML category builds models analysing similarities among input data, without requiring specific previous labels. Regarding supervised ML models, the most used in psychological research are Support Vector Machine (SVM), *k*th Nearest Neighbor (*k*NN), Alternating Decision Tree (ADTree), Artificial Neural Networks (ANNs), Recurrent Neural Networks (RNNs), Convolutional Neural Networks (CNNs), Random Forest (RF), Conditional inference Forest (CF), and Decision Trees (DT) (for a complete review of ML models used in psychological research see: Orrù et al., 2020; in ASD assessment see: Hyde et al., 2019). Such models can be seen as successful when they accurately predict target result, as well as when they can be generalized to new dataset. Data reduction removes irrelevant and redundant data, improving ML prediction, in particular in models like non-neural network approaches. Feature extraction methods allow to transform the input data space, preserving the most relevant information (Chumerin & Hulle, 2006). On the other hand, feature selection is the simplest method to reduce data dimensionality and it selects feature subset that maximise different objective functions, as for instance statistical differences. Feature extraction and selection can be applied either separately or in combination. To properly validate the ML algorithm performance on future data, cross-validation methods allow to separate the dataset into *n*-subset and to remove a subset from data before training, so that the model can be tested on that subset (Mello & Ponti, 2018). This process is reiterated until ML model has been trained on all data and the average among performance scores is computed. In a methodological perspective, cross-validation instances that guarantee good prediction outcomes are tenfold cross validation and leave-one-out cross-validation (Orrù et al., 2020). Several values aid researchers in interpreting ML results: accuracy is the percentage of correct prediction and it can be further reduced in sensitivity, that is the ability to correctly identify true positives, and specificity, that is the ability to correctly identify true negatives. Moreover, Cohen’s Kappa and the area under the curve (AUC) are further relevant values for the interpretation of ML results. The former relates the number of cases in each class to the number of cases in which the model has successfully matched the true class (Kappa values range from 0 to 1, where 0 represents a non-efficient model and 1 represents a perfect model), and the latter represents the area under the receiving operating character curve (that is a plot of sensitivity vs specificity), that reveals the ML method goodness in making categorical classifications.

In ASD research, ML has been involved for two purposes: pattern classification and stratification (Wolfers et al., 2019). Pattern classification represents the use of supervised models on either biological or behavioural measures in order to discern children with ASD from TD. Stratification refers to the application of unsupervised models on a variety of measures in order to define clusters in the ASD phenotype. ML has demonstrated its promising power for the objective ASD assessment on several measures, reporting classification accuracies between 60 and 98% (Wolfers et al., 2019). Some instances of measures used in the ASD assessment based on ML are EM analysis (e.g., Jiang & Zhao, 2017; Liu et al., 2016), body movements (e.g., Alcañiz Raya, Chicchi Giglioli, et al., 2020; Wu et al., 2018), and sensory processing (e.g., Alcañiz Raya, Giglioli, et al., 2020; Koirala et al., 2019). Regarding EM analysis, SVM classification on EM in a SI-based face recognition task provided an accuracy of 88.51% in the discrimination of children with ASD, with sensitivity of 93.10%, specificity of 86.21% and AUC of 0.8963 (Liu et al., 2016). ML models on EM towards more complex stimuli, as SVs, provided a classification accuracy of 85.1%, sensitivity of 86.5%, and specificity of 83.8% in the classification of children with ASD (Wan et al., 2019). In addition, several studies attempted to stratify ASD by the identification of clusters in the disorder phenotype (Wolfers et al., 2019). However, the use of EM measures in this field is not extended. Due to current ASD assessment limitations and the heterogeneity in disorder phenotypes, ASD assessment could benefit from ML, for both classification and stratification purposes, reducing diagnosis time and simultaneously improving accuracy (Hyde et al., 2019; Thabtah, 2019).

### Aim of the Systematic Review

Starting from these premises, the aim of this systematic review was to discuss the scientific evidence on early ASD classification based on SVA towards static and dynamic SS using ML techniques. More in detail, this systematic review contributes to the understanding of research on ASD social impairments, trying to discern which ML algorithms allow to discriminate children with ASD from TD peers. A complete discussion on ML approaches used in the attempt to distinguish children with ASD from TD peers, basing on their differences in SVA on SS and non-social stimuli is presented.

## Methods

Literature search followed the Preferred Reporting Items for Systematic Reviews and Meta-Analysis guidelines (PRISMA; Moher et al., 2009).

### Search Strategy

Studies were selected on July 6, 2020 from PubMed Central® and Scopus® database, searching for English peer-review articles, in which full-text was available, published after 2010. Search on database was conducted by the first author using the following Boolean string: ((ASD) OR (Autism)) AND ((eye movements) OR (gaze) OR (eye tracking) OR (eye-tracking)) AND ((children) OR (toddlers) OR (infants)) AND ((machine learning) OR (classification)) OR ((social stimuli) OR (social dynamic stimuli)). Studies which complied with the following inclusion criteria were selected: (a) patient group had a diagnosis of high/low functioning ASD; (b) control groups included at least a TD children sample; (c) experimental paradigms to measure SVA and non-SVA were presented on a display screen; (d) EM measures included fixations and saccades; (e) participants mean age range was 2–10 years old and sample must include at least 10 participants; (f) aim of the study was to discern children with ASD from TD peers using ML on EM data toward SS; (g) studies included not previously published data; (h) studies included randomized, control trials (RCTs). According to PRISMA recommendations on how to avoid the risk of bias, the four authors independently selected study abstracts and then evaluated full texts to check for the inclusion criteria. Relevant information which have been extracted from studies were the aim of the study, sample size and mean age, ASD assessment, eye-tracking stimuli, EM measures, data reduction technique, selected features, ML model, ML findings and conclusions.

## Results

### Review Flow

**Fig. 1:**
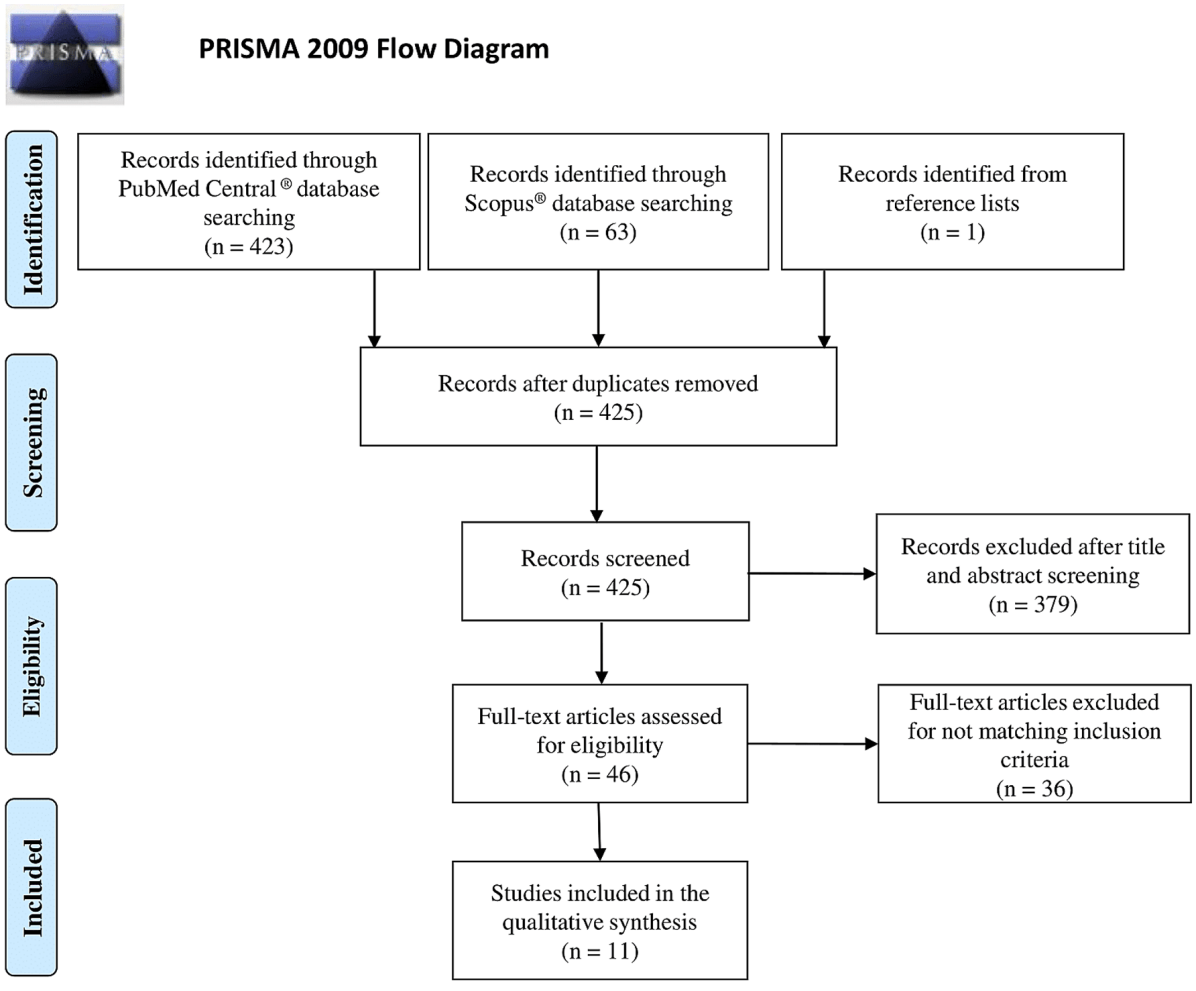
Flow diagram of study selection

The flow chart of the systematic review is shown in Fig. 1.

486 articles were found, respectively 423 in PubMed Central® and 63 in Scopus® database. Duplicated articles between database were removed (61 articles). Among remained 425 articles, 379 were excluded after title and abstract screening, and further 36 articles after full-text screening checking for inclusion criteria. Remained 10 articles plus 1 additional article from reference lists of selected articles met above criteria, with 11 articles included in the systematic review.

### Selected Studies of the Systematic Review

Selected studies are presented in Table 1 in alphabetic order.

**Table 1:** Selected studies

Authors	Mean age (SD)		Sample size (M)		Aim of the study	ASD assessment	Stimuli	EM measures	Data reduction	Selected features	ML model	ML findings	Conclusions
	ASD	TD	ASD	TD									
Carette et al., 2017	NR, age range of entire sample: 8–10		N = 17	N = 15	To detect ASD by the help of eye-tracking data and ML	NR	Dynamic	Duration, amplitude, acceleration, deceleration, and speed of saccades	No data reduction	EM measures of each participant	LSTM on EM measures	LSTM distinguished ASD from TD in 83% of tested patients	RNN can distinguish ASD from TD
			M NR	M NR			SV of a JA offer						
Carette et al, 2019	Entire sample: 7.88 (SD NR)		N = 29	N = 30	To help with ASD diagnosis	CARS	Dynamic	Fixation, saccade, blink, and EGC	Images scaling down, greyscale format conversion and eventually PCA	Eye gaze scanpaths	Non-neural network approaches (1);	(1) Generally, non-neural network approaches achieved AUC ≈ 0.7 (2) All ANN provided an A greater than 90%	ANN 1-Layer (200) achieved the best performance (A of 92%) and there was no substantial improvement growing the ML model complexity
			M NR	M NR			SVs and NSVs including NS elements and a presenter attempting a JA offer				Neural network approaches (2)		
Elbattah et al., 2019	Entire sample: 7.88 (SD NR)		N = 29	N = 30	To apply unsupervised ML to discover clusters in ASD EM	CARS	Dynamic	Fixation, saccade, blink, and EGC	Greyscale format conversion (1); PCA (2); t-SNE (3): Autoencoder (4)	Eye gaze scanpaths	12 k-means algorithm based on (1), (2), (3), and (4) and with different *k* values (from 2 to 4)	Poor separation of clusters in *k*-means algorithms based on (1), (2) and (3) *k*-means algorithm based on (4) yielded good quality of ASD/TD clusters and ASD severity clusters	There was a tendency of clustering structure in the dataset with faster eye gaze related to higher ASD severity that best emerged with (4) + *k*-means algorithm
			M NR	M NR			Same SS of Carette et al. (2019)						
Kang et al., 2020	4.29 (1.07)	4.26, (1.00)	N = 49	N = 48	To identify ASD using features from EEG and eye-tracking	Psychiatrists checking for DSM-V diagnostic criteria	Static	FDT	MRMR	Proportioned FDT in each AOI	SVM on selected features according to SS: (1), (2), and both types of faces (3)	(1) A 72.33%, AUC 0.8269	Among ML models on EM, the best model was (3), achieving an A of 75.89%
			39 M	36 M			SIs of girl face pictures of own-race (1) and other race (2)					(2) A 66.67%, AUC 0.7460	
												(3) A 75.89%, AUC 0.8652	
Li et al., 2018	NR, age range: 4–7	NR ,age range: 6–8	N = 53	N = 136	To automatically recognize ASD in raw video data	Psychiatrists checking for DSM-IV diagnostic criteria	Static	EGT	No reduction (1)	Holistic Acc H of EGT	SVM based on (1), (2), or (3) on different number of video frames	Best model was (3) + SVM on 40 video frames with an A of 93.7%	(3) + SVM on raw video data is a promising method to classifying ASD
			M NR	M NR			SIs of participant's mother		PCA (2)				
									KPCA (3)				
Li et al., 2020	Dataset 1 and 2: NR, age range: 4–7	Dataset 1 and 2: NR, TD age range: 6–8	Dataset 1: N = 53 M NR	Dataset 1: N = 136 M NR	To help early ASD assessment using deep learning on raw video data	Psychiatrists checking for DSM-V diagnostic criteria	Static	Angle and length of EGT	KPCA for SVM	Acc H and nAcc H of angle	SVM and LSTM models on both dataset using Acc H and nAcc H	Methods using Acc H outperformed nAcc H methods, and LSTM outperformed SVM. LSTM with fused Acc H on dataset 2 achieved the best A (92.6%)	LSTM outperformed SVM in ASD discrimination
			Dataset 2: Dataset 1 + 83 ASD (136) M NR	Dataset 2: Dataset 1 + 0 TD (136)			Same SS of Li et al. (2018)			Acc H and nAcc H of length			
										Combined angle and length Acc H and nAcc H			
Liu et al., 2015	7.85 (1.59)	TD-age = 7.73, (1.51)	N = 21 17 M	TD-age: N = 21 18 M	To propose an ASD prediction system based on ML techniques	AQ-Child	Static	EGC, EGT	*k*-means algorithm + N-Gram modelling (1), *k*-means algorithm + BoW (2), Predefined AOIs + BoW (3)	(1) Sequence of EGC	RBF kernel SVM on (1), (2), (3)	(1) AUC 0.5561, A of 72.13%	The two features are complementary to each other and ML model with fused features outperformed others ML models
							12 SIs depicting Chinese adult female faces			(2) BoW on EGC, BoW on eye motion, BoW on combined EGC and motion	In SVM on (2), EGC (4), eye motion (5) and both EGC and motion (6) were separately tested	(3) AUC 0.8208, A of 78.68%	
		TD-IQ = 5.69 (0.83)		TD-IQ: N = 20						(3) Face, nose, mouth, left eye and right eye		(4) AUC 0.8902, A of 81.97%	
				18 M								(5) AUC 0.9061, A of 85.25%	
												(6) AUC 0.9207, A of 86.89%	
Liu et al., 2016	7.90 (1.45)	TD-age = 7.86 (1.38)	N = 29	TD-age: N = 29 25 M	To examine whether face scanning patterns could be useful in ML-based ASD identification	AQ-Child	Static	Frequency distribution of face scanning coordinates without temporal information	*k*-means algorithm and histogram feature extraction	Same-race faces: 16	RBF kernel SVM on data from (1), (2), and all faces (3)	(1) A of 81.61%, AUC 82.40%	ML model on all faces achieved the best A in ASD discrimination from TD-age and TD-IQ
			25 M	TD-IQ: N = 29			6 SIs of faces of the same race (1) or other race (2)			Other-race faces: 64		(2) A of 90.80%, AUC 94.41%	
		TD-IQ = 5.74 (1.01)		25 M						All faces: 96		(3) A of 88.51%, AUC 89.63%	
Tao et al., 2019	8 (SD NR)	8 (SD NR)	N = 14	N = 14	To test whether combined CNN and LSTM can classify ASD	Psychiatrists checking for DSM-V diagnostic criteria	Static	Mean fixation duration, fixation count, and EGC	SalGAN and data pre-processing	Image patches of predicted saliency map based on individual scanpath	2 SP-ASDNet with different layer sizes: with batch normalization (1), and without (2)	Best model was (1) and it achieved an A of 74.22%	CNN-LSTM architecture can discriminate ASD from TD children
			M NR	M NR			300 SIs and NSIs						
Vu et al., 2017	NR, age range of entire sample: 2–10		N = 16	N = 16	To examine the impact of different SIs and exposure time on the screening A for ASD	ADOS	Static	Fixation maps	No data reduction	Gaze points in fixation maps	*k*NN on individual fixation maps on stimulus type (1), exposure time (2), and stimuli and duration combination (3)	Best models were: for (1) social scenes (A of 98.24%), for (2) 5 s (A of 95.24%), and for (3) social scenes for 5 s (A of 98.24%)	Social scene with full duration exposure (5 s) yielded the optimal result at nearly 100% of A
			M NR	M NR			12 SIs and NSIs related to social scenes, human faces, and object. SS had different exposure time (1, 3, 5 s)						
Wan et al., 2019	4.6 (0.7)	4.8 (0.4)	N = 37	N = 37	To develop an EM-based early diagnostic tool for ASD	Psychiatrists checking for DSM-V diagnostic criteria and CARS administration	Dynamic	FDT in each AOI	Permutation tests	Body and mouth AOIs	SVM on FDT in body and mouth AOIs	SVM achieved a classification A of 85.1%	Simple SVM model achieved same ASD classification A as more complex ET paradigms
			33 M	27 M			Short SV of a young Asian female mouthing the alphabet						

*A* accuracy, *Acc H* accumulative histograms method, *ADOS* Autism Diagnostic Observation Scale, *AQ-Child* Autism Spectrum Quotient: Children’s Version, *BoW* “Bag Of Words” features histogram representation, *CARS* Child Autism Rating Scale, *CNN* convolutional neural network, *EGC* eye gaze coordinates, *EGT* eye gaze trajectories, *FDT* fixation duration total time, *kNN* kth nearest neighbours algorithm, *KPCA* kernel principal component analysis, *LSTM* long short-term memory network, *MRMR* minimum redundancy maximum relevance method, *nAcc H* non-accumulative histograms method, *NR* not reported, *NS* non-social, *NSI* non-social image, *NSV* non-social video, *RBF* radial basis function, *SI* social image, *SS* social stimuli, *SV* social video, *TD-age* typical developmental group matched for chronological age, *TD-IQ* typical developmental group matched for IQ, *t-SNE* t-Distributed Stochastic Neighbor Embedding technique

Among the 11 studies, 10 used supervised ML models, whereas 1 used an unsupervised model (i.e., Elbattah et al., 2019). Among supervised ML models, 7 studies used SVM (Carette et al., 2019; Kang et al., 2020; Li et al., 2018, 2020; Liu et al., 2015, 2016; Wan et al., 2019), 3 used ANNs (Carette et al., 2017, 2019; Li et al., 2020), 1 used CNN-RNN architecture (e.g., Tao et al., 2019), 1 used *k*NN (Vu et al., 2017), and 1 used Naive Bayes and RF (Carette et al., 2019).

## Discussion

The aim of this systematic review was to present and discuss the ML models that were used to discern children with ASD from TD peers, through the measurement of EM towards static and dynamic SS. First, studies involving static SS were presented, and then studies that used dynamic SS. Liu et al. (2015) were the first who attempted to classify children with ASD from TD (N = 21) using EM on SIs depicting individual Chinese female faces. Features based on eye gaze coordinates, eye motion, and combined variables were extracted for each SI per subject using *k*-means clustering, creating a posteriori AOIs. Besides a posteriori AOI approach, traditional a priori AOI approach on combined variables was computed as baseline feature extraction method to compare performance of different SVM. Selected eye gaze coordinates and eye motion features were subsequently represented with N-Gram modelling and the orderless frequency Bag of Words (BoW). Five different SVM were trained: 3 using BoW histogram features on eye gaze coordinates, eye motion and combined variables after *k*-means clustering, 1 on eye gaze coordinates after N-Gram modelling, and 1 on selected features subsequent to a priori AOI definition. Leave-one-out strategy was used to train SVM on all participants except one, which was used as test set. The SVM performance based on BoW histogram features of combined variables derived from *k*-means clustering achieved the best performance with an AUC of 0.92 and an accuracy of 86.89% in ASD discrimination. In a subsequent study of the same authors (Liu et al., 2016), it was investigated the use of ML to classify EM of children with ASD and TD peers (N = 29) towards SIs depicting either individual Chinese faces or other race faces. Similar to the previous study, *k*-means clustering was used to define a posteriori AOIs, and histogram feature extraction provided feature representation per image for each subject. Different features were clustered for same race faces, other race faces and all faces. Leave-one-out cross-validation strategy was used to separate the training set from the test set and a radial basis function (RBF) kernel SVM for image-level classification was trained. Results provided evidence that the RBF kernel SVM on all faces outperformed the SVM on same race faces and other race faces, with an accuracy of 88.51%, sensitivity of 93.10%, specificity of 86.21%, and AUC = 0.89. In a like manner, Kang et al. (2020) tried to identify children with ASD (N = 49) from TD peers (N = 48) adopting supervised ML on features from two different measures: EEG and eye-tracking. EM were recorded using the same static SS of Liu et al. (2016), differently presented in terms of order and exposition time. SVM performance was tested with different inputs, which were respectively data from EEG measure, eye-tracking technique, and combined measures. Since the present review focused on EM toward SS in ASD, only related SVM were reported. Eight a priori AOIs were defined and results provided evidence of fewer gazes on face, nose, and mouth on both other and own-race faces for ASD. Feature selection was computed applying the minimum-redundancy-maximum-relevance method (Peng et al., 2005), which takes into account both minimum redundancy among features and maximum relevance with class labels, seeking the maximal statistical dependency between selected features. Classification accuracy of 3 SVM (own-race face, other-race face, both types of faces) were compared and best accuracy was achieved by the ML model related to both other and own-race faces, achieving an accuracy of 75.89% and AUC = 0.87. Overall, the best SVM performance was the one combining EEG and eye-tracking data, accomplishing an accuracy in ASD discrimination of 85.44% and AUC = 0.93, which suggests that multimodal assessment might lead to stronger results in the classification of ASD.

Aforementioned studies, which used similar SS and reduced sample size, seem to suggest that, on one hand, the use of combined variable features (i.e. eye gaze coordinates and motion; Liu et al., 2015) can enhance the ML performance, in relation to SVM based on features of single variables, and on the other, the use of features from combined stimuli (i.e., both same and other race faces; Kang et al., 2020; Liu et al., 2016) can provide better model accuracy rather than SVM based on just one type of SIs. In addition, Kang et al. (2020) found better accuracy when both eye-tracking and EEG data on both types of SIs were used to train SVM, emphasizing the importance of multimodal data acquisition in the early assessment of neurodevelopmental disorders as complex and varied as ASD. Li et al. (2018) took a previous dataset of raw videos of 53 children with ASD and 136 TD peers looking at their mother’s pictures on the screen, to later extract EM patterns in an indirect manner. The attempt was to develop an early ASD diagnosis tool based on raw videos that might be recorded at home rather than in clinics. Features as eye gaze trajectories were extracted from videos using the tracking learning detection algorithm. Inspired by the colour histogram method for images (e.g., Deng et al., 2001), the area of videos was divided into different zones and the number of eye displacements were counted within each zone. Due to the different methodology involved in the present study, neither a priori nor a posteriori AOI approach on the SS were applied. To ensure that integrity along the video timeline was preserved, the method of accumulative histograms was introduced and authors computed the ASD and TD holistic accumulative histograms based on different numbers of video frames (i.e., 20, 40, 50, and 100). SVM was chosen as ML algorithm to test the ability of the video-based method to discriminate ASD. To assess the efficacy of the method based on accumulative histograms, it was compared to a baseline method and a similar method based on histograms including all video frames (i.e., Torii et al., 2016). Principal component analysis (PCA) and kernel PCA (KPCA) were applied to extract features and therefore reduce data dimension. Sixteen SVM were fed and 20-fold cross validation was used to avoid overfitting issue. Although all models achieved an accuracy greater than 77%, highest accuracies were obtained using KPCA, and the best model was SVM on 40 video frames with an accuracy of 93.7%. However, the TD group was three times bigger than the ASD group, hence the imbalanced sample affected the ML accuracy score, which tended to be biased towards the sample group with more elements (Nguyen et al., 2009). To overcome the unbalanced sample issue, Li et al. (2020) used the same dataset of the previous study and a further new dataset of similar raw videos of children with ASD (N = 83). The new dataset was a balanced dataset of 272 raw videos (i.e., dataset 2) in which participants’ face was recorded while they were looking at their mother’s picture on the screen. As in the previous study, AOI could not be outlined due to the type of data and eye gaze trajectories on SIs were computed using the tracking learning detection algorithm and then divided into angle and length features. Accumulative and non-accumulative histograms were then generated for single and combined features with the intention to use them as inputs for neural networks. Neural networks mimic the human brain functioning, receiving data as input and providing an output previously defined by the operator among prediction, classification and correlation. Several neural network models have been developed: ANN, RNN, and more complex and robust RNN as long short-term memory (LSTM; Hochreiter & Schmidhuber, 1997). LSTM implementation follows the RNN one, except for some nodes, as an additional one that is used as memory rubber and it is fed by a forget gate. Six LSTM networks were trained using tenfold-cross validation with respectively non-accumulative and accumulative histograms of single and combined variables. In addition, KPCA to reduce data was computed in order to feed 6 SVM and compare their performance to LSTM performance. Results revealed that features based on accumulative histograms yielded better outcomes than non-accumulative histograms, and LSTM networks outperformed SVM by 6.2% in accuracy. The best performance in ASD discrimination was achieved by LSTM with combined accumulative histograms on dataset 2 (accuracy of 92.60%, sensitivity of 91.9%, and specificity of 93.4%). LSTM is usually more efficient with data providing time dimension rather than with orderless data, representing in this case a well-fit solution in relation to SVM. However, even though sample size was improved in relation to Li et al. (2018), providing more validity to the ML models, EM were indirectly measured, since they were extracted from raw videos of participants looking at SS at their houses, rather than directly recorded using an eye-tracking system. The purpose of these studies was to develop a simple cost-effective tool for home as rapid ASD screening, and the involvement of a portable eye-tracker for the direct EM recording could enhance the feasibility of the method. Moreover, involved SS differed between participants, since each child looked at the picture of his or her mother, increasing EM variability. The presence of controlled setting, direct EM measurement, and same SS for participants might improve both accuracy and objectivity of the method.

Tao et al. (2019) integrated CNN and LSTM to classify children with ASD and TD basing on their scanpaths related to 300 SIs and non-social images (NSIs) presenting either people or objects and naturalistic scenes. Scanpaths are considered as visual representations describing EM dynamics on stimuli, such as the sequence of fixations and saccades (Goldberg & Helfman, 2010). EM dataset was the Saliency4ASD grand challenge, which is an EM dataset publicly released to evaluate ASD classification algorithms gathering EM data of 14 TD and 14 children with ASD (Duan et al., 2019). Starting from fixation points, the reference saliency map was created using the neural network SalGAN (Pan et al., 2017) and features were extracted from the patches related to eye gaze coordinates in the saliency map. Subsequently, extracted features were given as input to two CNN-LSTM architectures, which differed in the number of layers. The best performance in ASD discrimination was achieved by the CNN-LSTM architecture of 6 layers with batch normalization (accuracy of 74.22%). Batch normalization is a technique that improves both ANN speed and performance, normalizing the input layer by re-centering and re-scaling (Ioffe & Szegedy, 2015). In accordance with Li et al. (2020) and Tao et al. (2019), RNN show potential as algorithms that can be used to automatically assess ASD involving static SS. However, Tao et al. (2019) tested CNN-LSTM architectures on a reduced amount of data and the increase of sample size might enhance the strength of the RNN model. As discussed above, the use of combined variables, such as eye gaze coordinates and trajectories, or mean fixation duration and fixation counts, seem to improve accuracy in both SVM and RNN (e.g., Li et al., 2020; Liu et al., 2015; Tao et al., 2019). Vu et al. (2017) studied the combined effect of different SIs and exposure time on the accuracy of ML-based assessment of ASD. Their purpose was to look for the SS and exposure time that yielded the best ASD discrimination accuracy. Children with ASD and TD (N = 16) looked at SIs of social scenes and paired human faces and to NSIs of objects, both presented on the screen for different exposure times (1, 2, or 5 s). Participants’ fixation distribution maps on each stimulus were computed using *k*NN, a learning algorithm which saves all data instances in *n*-dimensional space. New data classification is based on the classification of the closest *k* number of stored instances (Orrú et al., 2020). In this case, *k* was determined as 3. In each image, one fixation from the computed fixation maps was chosen and remaining gaze points were used to train *k*NN, which was applied 30 times sequentially. *f*-score accuracy was computed for each image, as an average among *k*NN accuracies. The most complex SI representing social scenes achieved the best accuracy in ASD and TD discrimination (98.24%) whereas among exposure times 5 s yielded the best accuracy (95.24%). Finally, SS and exposure time combinations provided evidence that shorter exposure times were weak and not recommended, whereas the best model was the one presenting social scene for 5 s, with an accuracy of 98.24%. Despite the reduce sample size, Vu et al. (2017) findings were in line with Chita-Tegmark (2016) and Frazier et al. (2017) meta-analyses, since the best accuracy was achieved by the most complex SS presented for the longest time. In the majority of studies presented so far, the combined use of distinct elements in static SS, such as faces related to different races, social and non-social elements, or embellished social scenes, improved ML accuracy in relation to models based on the same uncombined elements (e.g., Kang et al., 2020; Liu et al., 2016; Tao et al., 2019; Vu et al., 2017). In addition, further improvements in the ability of ML algorithm to discern ASD from TD were provided by the combination of features from different dependent variables (e.g., Li et al., 2020; Liu et al., 2015) and by the multimodal acquisition of data (e.g., Kang et al., 2020).

Wan et al. (2019) and Carette et al. (2017, 2019) are the three studies that involved dynamic SS and supervised ML. In Wan et al. (2019) participants (N = 37) watched a video of a young Asian female mouthing the alphabet. In order to estimate a priori AOI reliability for ASD classification, AOI discrimination weights were tested using permutation tests. SVM with fivefold-cross validation on fixation time on AOIs with discriminative power was computed and the accuracy in ASD classification achieved 85.1%, with a sensitivity of 86.5%, and a specificity of 83.8%. Carette et al. (2017) wanted to use a neural network approach to discern between children with ASD (N = 17) and TD (N = 15), according to their EM toward SV presenting a JA offer. Since neural networks can manage high data dimension, no data reduction was applied. EM measures on the SV without outlining AOIs were considered. Chosen RNN was two LSTM hidden layers of 20 neurons each using different fitness values. LSTM provided promising results, achieving to correctly classify 5 subjects out of 6 of the test set (i.e., accuracy of 83%), with a confidence greater than 95%. However, sample size was reduced and the amount of training data (4 children with ASD and 3 TD) was small for a RNN that can manage broader dataset for training. The involvement of much more participants could have reduced both uncertainty and overfitting. In a subsequent study, Carette et al. (2019) recorded EM of children with ASD (N = 29) and TD (N = 30) on dynamic SS to apply several ML algorithms. Involved SS were SVs representing a JA offer toward the unique object placed around, and NSVs with attractive elements for children, such as colourful balloons and cartoons. EM were recorded and later computed to draw individual scanpaths labelled according to sample groups, which avoided the AOI analysis. Due to the small number of generated scanpaths, image augmentation was applied producing synthetic samples by image transformation operations (e.g., rotation) in order to reduce uncertainty and to improve accuracy. The new dataset was five time bigger than the original one. To reduce data dimension, all images were firstly scale down, then converted to greyscale, and finally PCA was eventually applied. Traditional ML approaches were fed with selected features and tested for ASD discrimination. Involved ML models were Naïve Bayes, SVM, and RF. Moreover, ANN were implemented and tested for the same purpose. Developed ANN included single hidden layer of 50 neurons, 200 neurons, and 500 neurons as well as 2 hidden layers with respectively 80 and 40 neurons. Ten-fold-cross validation was applied. Outcomes revealed that more traditional ML approaches achieved on average an AUC of 0.7, as opposed to ANN, which provided accuracies greater than 90%. In particular, the single layer model of 200 neurons achieved the best performance in ASD discrimination (accuracy of 92%), suggesting that increasing the ANN complexity did not provide better results. The synthetic production of scanpaths nonetheless, allowed the computation of robust ANN, which would have been weaker if they were based just on the real sample size. Finally, there is only one study that used unsupervised ML on ASD EM (Elbattah et al., 2019). The aim of the study was to stratify ASD, in order to discover ASD EM clusters related to the disorder symptom severity. Children with ASD (N = 29) and TD (N = 30) looked at same SS of Carette et al. (2019). EM were recorded and individual scanpaths were created and scaled down for dimension reduction, avoiding AOI analysis. In order to test which combination between feature extraction methods and *k*-means algorithm might provide better outcomes in the stratification of ASD based on EM, four feature extraction methods were compared: converting scanpaths into grayscale, PCA, the t-Distributed Stochastic Neighbor Embedding technique (t-SNE; Maaten & Hinton, 2008), and the autoencoder, which is a particular unsupervised ANN. *k*-means algorithm was then used to develop 4 clustering models based on different features derived by feature extraction methods. Three clustering structures were studied, as represented by selected *k* values (*k* = 2, *k* = 3, and *k* = 4). Results showed that the quality of clusters decreased increasing *k* value, and clusters separation using pixel-based features, PCA, or t-SNE was poor, as opposed to autoencoder that provided better cluster quality with faster EM related to higher ASD symptom severity.

Among the few studies that involved ML algorithms and dynamic SS, Wan et al. (2019) was the only one applying SVM, whereas Carette et al. (2017, 2019) opted for supervised ANN, and Elbattah et al. (2019) for unsupervised ANN. Taking into account studies that involved dynamic SS and ML for ASD classification rather than stratification (e.g., Carette et al., 2017, 2019; Wan et al., 2019), dynamic SS varied from SV of one actress, to complex SVs presenting JA offers eventually combined with NSVs. SVs can be considered as dynamic SIs, since they are composed by a myriad of static frames depicting social elements. In line with findings of selected studies involving static SS with combined social elements (e.g., Kang et al., 2020; Liu et al., 2016; Tao et al., 2019; Vu et al., 2017), dynamic SS such as SVs represent complex stimuli full of details, that can be promising for the discrimination of ASD. In addition, the use of data related to complex SVs, as well as to the combination of SVs and NSVs might further improve ML models, due to the greater SS complexity and the variability in recorded data. However, although Carette et al. (2017, 2019) and Wan et al. (2019) achieved accuracies greater than 80% in ASD discrimination using dynamic SS, sample sizes were small, in particular when ANN were involved. Only Carette et al. (2019) tried to overcome this issue by creating synthetic samples, reducing uncertainty and overfitting.

### Overall Findings

In summary, the majority of studies applied as data reduction technique features extraction rather than features selection, due to the broad feature dimensionality, which is related to the presence of many dependent variables rather to greater sample sizes. The majority of studies indeed reported small sample sizes which occasionally needed data augmentation by the computation of synthetic samples (e.g., Carette et al., 2019), improving strength and accuracy of the ML model. Regarding AOIs, just few studies used a posteriori AOI approach (e.g., Liu et al., 2015, 2016), whereas the majority of studies either did not consider AOIs (e.g., Carette et al., 2017, 2019; Elbattah et al., 2019; Tao et al., 2019; Vu et al., 2017) or based the analysis on a priori AOIs (e.g., Kang et al., 2020; Li et al., 2018, 2020; Wan et al., 2019). AOIs may be irrelevant in some studies, in particular when the analysis is based on approaches that convert data, such as scanpath. However, whether the analysis requires AOIs, we suggest to consider a posteriori AOI approach, since it provides a data-driven selection of relevant areas in the SS. Despite Elbattah et al. (2019) that involved unsupervised ANN in the attempt to stratify ASD, remaining studies used typical supervised ML algorithms to predict and classify ASD. Studies involving static SS, which are the majority, used *k*NN, SVM and RNN, whereas studies involving dynamic SS assessed the performance of SVM, ANN, and RNN. The combination of different elements (i.e., dependent variables, stimuli) when static SS were involved seemed to enhance SVM performance, providing stronger results in the classification of ASD. Similarly, dynamic SS, due to the greater amount of details and the presence of both animation and combined social elements, can enhance the ability of ML algorithm to discriminate ASD. Overall ML algorithms achieved a fair classification accuracy greater than 80%, except for Tao et al. (2019), and Kang et al. (2020) which achieved 75.89% accuracy involving only eye tracking data. Nevertheless, the combined features from eye tracking and EEG data in Kang et al. (2020) gained an accuracy of 85.44%, suggesting that multimodal assessment increased accuracy and hence reliability of the assessment process.

## Conclusions and Future Directions

The aim of this systematic review was to discuss the recent scientific evidence on ML models used to classify children with ASD and TD according to their EM on different SS (i.e., static and dynamic). Along with the traditional ASD assessment, which represents a qualitative method to diagnose ASD, ML and EM-based procedures might fulfil the need for quantitative method in the ASD diagnosis. However, on one hand, studies tended to involve small sample sizes, which affected the reliability of discrimination accuracy in ML models, and on the other, they mostly involved static SS rather than dynamic SS. Both SVM and neural networks achieved interesting results in the ASD classification, but SVM seems to be more promising and cost-effective, since it is efficient even with less sample cases, it requires fewer amount of parameters to be set for training, and less computational cost. It might be interesting testing further ML models, such as ADTree, DT, and RF, in order to validate a unique ML approach for the assessment of ASD (Thabtah, 2019). Similarly, due to the complexity in the ASD phenotype, as well as the comorbidity with other diseases (e.g., ADHD), it might be also interesting attempting to classify ASD through bottom-up processes as unsupervised ML, which so far have been mostly used in ASD stratification rather than classification (Wolfer et al., 2019). Concerning the second issue of the traditional ASD assessment, which is the lack of ecological validity, previous works not involving ML presented dynamic SS as possible solution, since they are more naturalistic than static SS (e.g., Cilia et al., 2019a, 2019b; He et al., 2019). Accordingly, selected studies suggested the preferential use of dynamic SS over static SS, due to the greater complexity and the presence of combined social elements. Dynamic SS nonetheless diverge from realistic settings for many aspects, and the involvement of controlled and standardized procedures, based on new technologies, might definitely overcome this ecological validity issue. New technologies indeed, such as virtual reality (VR), have already proven their power in both ASD diagnosis and intervention (Parsons, 2016; Parsons, 2016), providing cost-effective realistic situations that strongly represent real life and allow to control the environment wherein is safe to test children with ASD. In particular, studies with semi-immersive VR system (i.e., CAVE™) involving several implicit measures disclosed promising results in the discrimination of ASD (Alcañiz Raya, Chicchi Giglioli, et al., 2020; Alcañiz Raya, Giglioli, et al., 2020). Along with the traditional ASD assessment, multimodal VR-based assessment involving ML procedures and several implicit measures such as EDA, body movements, and EM, which objectively tap ASD dysfunctions reported in DSM V (APA, 2013), can contribute to the development of a more objective and ecological method for the ASD early diagnosis.

## Declarations

### Research Involving Human and Animal Participants

The study did not involve human participants and/or animals.
